# *FCGR3A* gene duplication, FcγRIIb-232TT and FcγRIIIb-HNA1a associate with an increased risk of vertical acquisition of HIV-1

**DOI:** 10.1371/journal.pone.0273933

**Published:** 2022-09-09

**Authors:** Joy Ebonwu, Ria Lassaunière, Maria Paximadis, Renate Strehlau, Glenda E. Gray, Louise Kuhn, Caroline T. Tiemessen

**Affiliations:** 1 Division of Public Health Surveillance and Response, National Institute for Communicable Diseases, Johannesburg, South Africa; 2 Faculty of Health Sciences, University of the Witwatersrand, Johannesburg, South Africa; 3 Department of Virus and Microbiological Special Diagnostics, Virus Research and Development Laboratory, Statens Serum Institut, Copenhagen, Denmark; 4 Centre for HIV & STIs, National Institute for Communicable Diseases, Johannesburg, South Africa; 5 Empilweni Services and Research Unit, Rahima Moosa Mother and Child Hospital, Johannesburg, South Africa; 6 Department of Paediatrics and Child Health, Faculty of Health Sciences, University of the Witwatersrand, Johannesburg, South Africa; 7 Perinatal HIV Research Unit, Faculty of Health Sciences, University of the Witwatersrand, Johannesburg, South Africa; 8 South African Medical Research Council, Cape Town, South Africa; 9 Department of Epidemiology, Gertrude H. Sergievsky Centre, Vagelos College of Physicians and Surgeons, Mailman School of Public Health, Columbia University Irving Medical Center, New York City, New York, United States of America; Unicamillus, Saint Camillus International University of Health Sciences, ITALY

## Abstract

**Background:**

Some mother-to-child transmission (MTCT) studies suggest that allelic variations of Fc gamma receptors (FcγR) play a role in infant HIV-1 acquisition, but findings are inconsistent. To address the limitations of previous studies, the present study investigates the association between perinatal HIV-1 transmission and FcγR variability in three cohorts of South African infants born to women living with HIV-1.

**Methods:**

This nested case-control study combines *FCGR* genotypic data from three perinatal cohorts at two hospitals in Johannesburg, South Africa. Children with perinatally-acquired HIV-1 (cases, n = 395) were compared to HIV-1-exposed uninfected children (controls, n = 312). All study participants were black South Africans and received nevirapine for prevention of MTCT. Functional variants were genotyped using a multiplex ligation-dependent probe amplification assay, and their representation compared between groups using logistic regression analyses.

**Results:**

*FCGR3A* gene duplication associated with HIV-1 acquisition (OR = 10.27; 95% CI 2.00–52.65; *P* = 0.005) as did the FcγRIIb-232TT genotype even after adjusting for *FCGR3A* copy number and *FCGR3B* genotype (AOR = 1.72; 95%CI 1.07–2.76; *P* = 0.024). The association between FcγRIIb-232TT genotype and HIV-1 acquisition was further strengthened (AOR = 2.28; 95%CI 1.11–4.69; *P* = 0.024) if adjusted separately for *FCGR2C* c.134-96C>T. Homozygous FcγRIIIb-HNA1a did not significantly associate with HIV-1 acquisition in a univariate model (OR = 1.42; 95%CI 0.94–2.16; *P* = 0.098) but attained significance after adjustment for *FCGR3A* copy number and *FCGR2B* genotype (AOR = 1.55; 95%CI 1.01–2.38; *P* = 0.044). Both FcγRIIb-232TT (AOR = 1.83; 95%CI 1.13–2.97; *P* = 0.014) and homozygous FcγRIIIb-HNA1a (AOR = 1.66; 95%CI 1.07–2.57; *P* = 0.025) retained significance when birthweight and breastfeeding were added to the model. The common *FCGR2A* and *FCGR3A* polymorphisms did not associate with HIV-1 acquisition.

**Conclusions:**

Collectively, our findings suggest that the FcγRIIb-232TT genotype exerts a controlling influence on infant susceptibility to HIV-1 infection. We also show a role for less studied variants–*FCGR3A* duplication and homozygous HNA1a. These findings provide additional insight into a role for FcγRs in HIV-1 infection in children.

## Introduction

Antibody crystallisable fragment (Fc) gamma receptors (FcγRs) are hematopoietic cell surface glycoproteins that bind the Fc region of immunoglobulin G (IgG) antibodies, linking both humoral and cellular branches of immunity. Cross-linking of FcγRs on the cell surface initiates and regulates immune mechanisms that include antibody-dependent cellular cytotoxicity (ADCC), antibody-dependent cellular phagocytosis (ADCP), antibody production, B-cell activation, antigen presentation, and cytokine production [1–4]. Cumulative data have highlighted the role of Fc-mediated effector functions in human immunodeficiency virus 1 (HIV-1) acquisition and post-infection control of viremia [2, 5–13].

Generally, FcγRs are divided into three classes (FcγRI, FcγRII, and FcγRIII), each with different isoforms and encoded by different genes. The classes differ in structural domain organisation, affinity for specific IgG subclasses and ability to trigger activating or inhibitory signals [14, 15]. While FcγRI binds monomeric IgG with high affinity, both FcγRII and FcγRIII bind to IgG complexes through multivalent and low affinity interactions [16]. The low affinity FcγRs located on chromosome 1q23 (FcγRIIa, FcγRIIb, FcγRIIc, FcγRIIIa and FcγRIIIb) are encoded by *FCGR2A*, *FCGR2B*, *FCGR2C*, *FCGR3A and FCGR3B* genes, respectively [15] and they play different roles in regulating immune responses [4].

Functionally-relevant genetic variants, including single nucleotide polymorphisms (SNPs) and copy number variation (CNV), have been characterized in low-affinity receptors and associated with different diseases [10, 12, 15, 17–22]. Generally, CNV is considered an important factor of inter-individual differences and to date, CNV has been demonstrated for only *FCGR2C*, *FCGR3A* and *FCGR3B* [23, 24]. Variation in copy number of *FCGR3A* correlates with FcγRIIIa surface expression levels on natural killer (NK) cells, a key mediator of ADCC [23]. Similarly, CNV of *FCGR3B* directly correlates with protein expression and uptake of immune complexes by neutrophils [25]. Functionally-significant amino acid changes have been reported for FcγRIIa, FcγRIIb, FcγRIIIa and FcγRIIIb that affect either their binding affinity for IgG or receptor function. An arginine (R) to histidine (H) substitution at amino acid position 166 of FcγRIIa (position 131 in the mature protein), alters the receptor’s affinity of IgG and its subclasses. In FcγRIIb, an isoleucine (I) to threonine (T) substitution at position 232 in the full protein reduces its inhibitory function on B cells [15]. A polymorphism in FcγRIIIa results in a substitution of valine (V) to phenylalanine (F) at amino acid position 176 (position 158 in the mature protein) that alters the receptor’s affinity for IgG and its subclasses [15, 26]. Conversely, a combination of five amino acid changes in FcγRIIIb give rise to the human neutrophil antigen 1 (HNA1) variants, HNA1a, HNA1b and HNA1c. Neutrophils from HNA1a homozygous individuals display greater phagocytic capacity compared to HNA1b homozygous individuals [27].

Accumulating evidence from mother-to-child transmission (MTCT) studies suggests that allelic variations of FcγR play a role in infant HIV-1 acquisition, but the observed results are inconsistent [11, 12, 28]. Specifically, Brouwer et al. reported a positive association between the FcγRIIa 166HH genotype and perinatal HIV-1 acquisition in a cohort of infants in Kenya [11] that was not observed in subsequent separate studies in Kenya and South Africa [12, 28]. The functional consequence for FcγR variants beyond FcγRIIa-H166R and FcγRIIIa-F176V during HIV-1 infection and acquisition *in vivo* has not been largely investigated. Further studies are therefore needed to elucidate the definitive role of *FCGR* genotypes on MTCT of HIV-1.

The potential role of FcγR-mediated effector functions in modulating perinatal HIV-1 transmission and acquisition was investigated for the first time in South Africa, using FcγR variants as proxy for functional capacity [12]. The study differed from the those conducted in Kenya [11, 28] in that it investigated variation at multiple loci and gene copy number variation in the *FCGR* locus. The population differences within Africa warrants that genetic association studies are done for specific populations. The study found the maternal FcγRIIIa-158V allele, which confers enhanced antibody binding affinity and ADCC capacity, to be significantly associated with reduced HIV-1 transmission. In both mother and infant, having an FcγRIIIb-HNA1b allotype (that reduces neutrophil-mediated effector functions) was associated with increased HIV-1 transmission and acquisition, respectively. On the other hand, homozygosity for the FcγRIIIb-HNA1a allotype in the infant was protective of perinatal HIV acquisition. Since FcγRIIIb is largely expressed in neutrophils, the study findings were suggestive of a potential role for neutrophils in modulating perinatal HIV-1 transmission and acquisition. However, a relatively small number of HIV-1 infected infants (n = 78) were genotyped. This study further interrogates the role of FcγR-mediated effector functions in modulating perinatal HIV-1 acquisition, using a much larger cohort. As we strive towards the goal of elimination of vertical HIV-1 transmission, more studies are required to elucidate natural mechanisms of protection in order to identify novel targets for preventative and therapeutic interventions.

## Materials and methods

Ethics approval for the study was obtained from the University of the Witwatersrand Human Research Ethics Committee (Reference numbers: M170585; M180575). Written informed consent to participate in this study was provided by the participants’ legal guardian/next of kin.

### Study design and population

A nested case-control study was carried out to assess the association between low affinity *FCGR* variability and HIV-1 perinatal acquisition in children, combining data from past studies of three perinatal cohorts at two hospitals in Johannesburg, South Africa [29–32]. The HIV-1-infected cohort (cases) consists of 546 children who were recruited as part of two sequential randomized clinical trials (NEVEREST 2 and 3) [29–31]. The remaining two cohorts comprised of 566 HIV-1-exposed uninfected children (controls) [32]. For this study, only available samples with sufficient material were genotyped. *FCGR* genotypic data from 395 HIV-1-infected children were compared with 312 of the HIV-1-exposed uninfected children. All study participants were black South Africans and received nevirapine for prevention of MTCT. The receipt of nevirapine at birth, known to significantly reduce intrapartum transmission [32–34], suggests that most of the infants were likely infected in utero. Maternal antiretroviral therapy was not routinely used at the time. The available demographic and perinatal variables included for both cases and controls are sex, birthweight, breastfeeding status and gestation (term or pre-term).

### Genotyping

Functional *FCGR* variants were genotyped using the *FCGR*-specific multiplex ligation-dependent probe amplification assay (MRC Holland, Amsterdam, The Netherlands) according to manufacturer’s instructions [18, 35]. In two reactions, the assay detects genomic copy number of *FCGR2C*, *FCGR3A* and *FCGR3B*, as well as functional allelic variants: FcγRIIa-H166R (alias H131R), FcγRIIb-I232T, FcγRIIIa-V176F (alias V158F) and FcγRIIIb-HNA1a/b/c. Furthermore, the assay detects *FCGR2C* SNPs that affect gene expression– c.169T>C (p.X57Q), c.798+1A>G, and the *FCGR2B/C* promoter variant at position c.−386G>C and c.−120A>T. Amplicons were separated by capillary electrophoresis on an ABI Genetic Analyser 3130 (Life Technologies, Applied Bio systems, Foster City, CA, USA) and fragments analysed with the Coffalyzer.NET software (MRC Holland) using peak height as a measure of gene/allele copy number. In this study, we did not distinguish *FCGR2B* and *FCGR2C* promoter sequences since earlier findings indicate that African individuals do not possess the promoter variant in *FCGR2B*, and thus any detected c.−386G>C minor alleles would be in *FCGR2C* (37). The SNP nomenclature used in this manuscript refers to positions in accordance with the Human Genome Variation Society (HGVS) guidelines [36]. The numbering of nucleotides is relative to the Genome Reference Consortium Human Reference 38 [GRCh38 (hg38)].

### Statistical analysis

Categorical data were presented as absolute numbers and percentages. The Chi-squared and Fisher Exact tests (where appropriate) were used for comparisons between children with HIV-1-infection and children who were HIV-1-exposed uninfected. Univariate and multivariate logistic regression analyses were conducted to determine the association between functional *FCGR* variants and perinatal HIV-1 acquisition. Each *FCGR3B* genotype is defined as the combination of FcγRIIIb-HNA1a/b/c allotypes present or absent irrespective of gene copy number. Genotype reference groups for the di-allelic FcγRIIa-H166R, FcγRIIb-I232T, and FcγRIIIa-V176F variants were homozygosity for the major allele, while the genotype reference group for the multi-allelic FcγRIIIb-HNA1a/b/c were selected based on prevalence. A *P* value *<* 0.05 in the multivariate analysis was regarded as statistically significant and 95% confidence intervals (CI) were used to estimate precision. Adjustment for multiple comparisons was performed using the Bonferroni correction, which considered six independent tests for the different variants—*FCGR3A* copy number, *FCGR3B* copy number, FcγRIIa-H166R, FcγRIIb-I232T, FcγRIIIa-V176F and FcγRIIIb-HNA1a/b/c. Both unadjusted and adjusted *P* values are reported. All analyses were performed in STATA version 15.1 (StataCorp LP, Texas, USA).

Linkage disequilibrium (LD) between functional *FCGR* variants was assessed using the Haploview software package [37] and expressed as D prime (D′) and square of the correlation coefficient (r^2^). The closer D′ is to 1 the stronger the LD between two loci. We assessed LD for FcγRIIIb-HNA1a/b/c allotype using tag SNP p.N65S (amino acid change from asparagine to serine at position 65) that differentiates HNA1a (P.65N) from HNA1b|c (p.65S), and the SNP that differentiates HNA1b from HNA1c, resulting in aspartic acid replacing alanine at amino acid position 78 (p.A78D). Genotypic data with multiple gene copies were considered homozygous if all copies carried the same allele or heterozygous when both alleles were present. Hardy-Weinberg equilibrium was considered for individuals with two gene copies and the statistics abstracted from the Haploview analysis output.

## Results

### Study population characteristics

There were no significant differences in sex and gestation between the 395 HIV-1 infected (cases) and 312 HIV-1-exposed uninfected (controls) included in this analysis. However, a higher proportion of HIV-infected children had a low birth weight (<2500g; *P* <0.001) and were breastfed (*P* <0.001) than controls (Table 1).

**Table 1:** Characteristics of perinatal HIV-1 acquisition in our study cohort.

Characteristics	HIV-1-exposed uninfected	HIV-1 infected	*P* value
	n = 312	n = 395	
Sex			0.661
Male	160 (51)	196 (49.6)	
Female	152 (49)	199 (50.4)	
Gestation	(n = 312)	(n = 389)	0.180
Term	282 (90)	339 (87)	
Preterm (<37 weeks)	30 (10)	50 (13)	
Birth weight (g)	(n = 312)	(n = 375)	**<0.001**
≥ 2500	282 (90)	295 (79)	
< 2500	30 (10)	80 (21)	
Breastfed	(n = 311)	(n = 388)	**<0.001**
No	289 (93)	297 (77)	
Yes	22 (7)	91 (23)	

Data are expressed as n (%).

Total numbers analyzed for each variable are indicated.

Bold indicates statistical significance of *P <* 0.05.

### Distribution of *FCGR* copy number variation and HIV-1 acquisition

**Fig 1:**
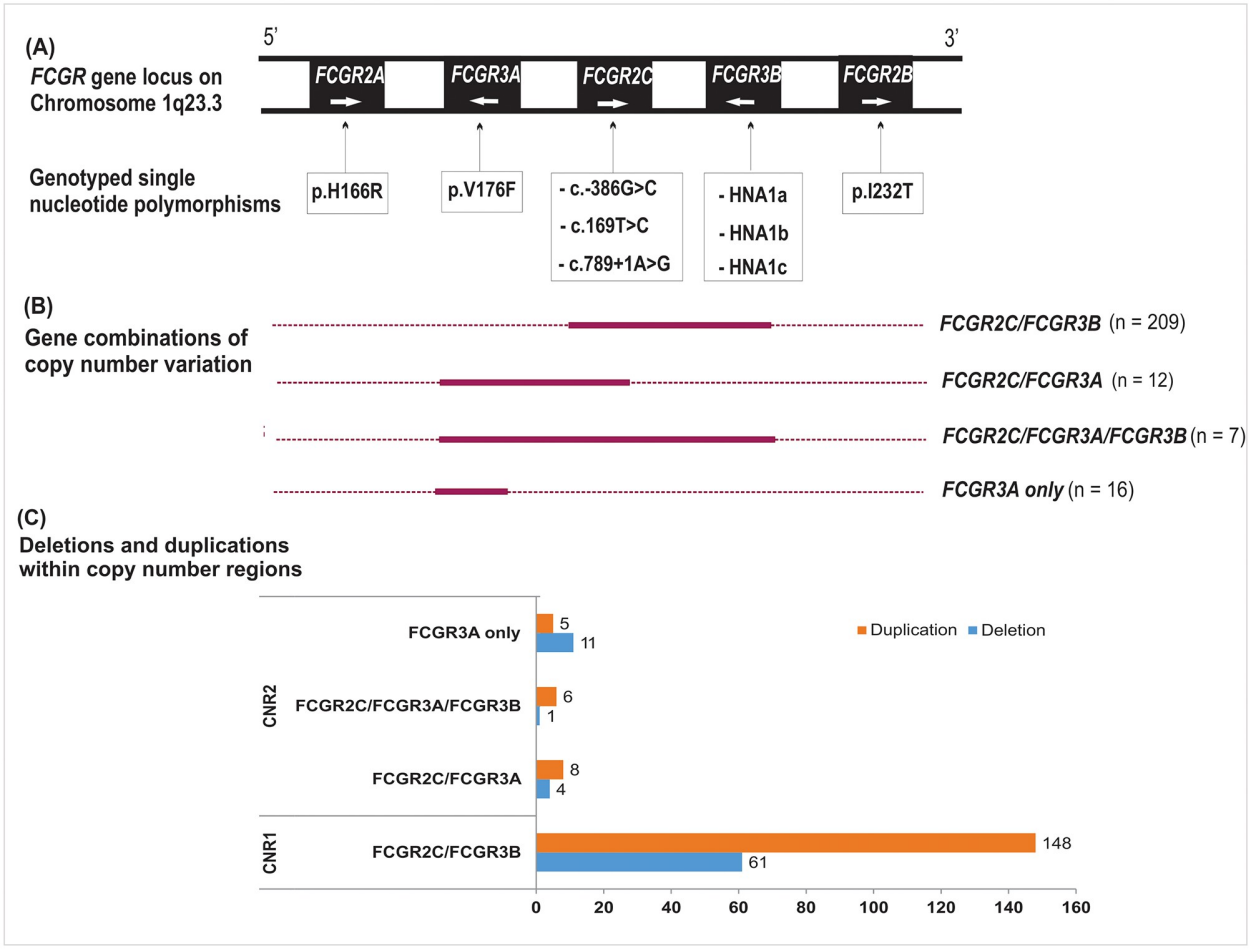
Diagrammatic representation of the FCGR2/3 locus structure and variation. **(A)** The *FCGR2* and *FCGR3* genes on human chromosome 1q23 with their orientation and the functional polymorphisms genotyped in the study. Polymorphic amino acids are indicated by one-letter code. **(B)** CNV has been previously described within distinct copy number variable regions (CNRs) [38, 39]. Four gene combinations of CNV, either duplication or deletion, were observed and are indicated as solid lines. The *FCGR2C/FCGR3B* and *FCGR2C/FCGR3A* combinations correspond to the previously designated CNR1 and CNR2, respectively. **(C)** Copy number region deletions and duplications within CNR1 and CNR2. This displays further breakdown of individuals with either a deletion or duplication within the 4 distinct gene combinations of CNV.

Genes are deleted or duplicated at the *FCGR2/3* locus within previously defined copy number variable regions (CNRs) [38–40]. Fig 1 shows the SNPs genotyped (A) and the 4 distinct patterns of CNV in the present South African cohort: *FCGR2C/FCGR3B*, *FCGR2C/FCGR3A*, *FCGR2C/FCGR3A/FCGR3B* and *FCGR3A* only (Fig 1B). The most common variation was observed for the combined duplication/deletion of complete *FCGR2C* and *FCGR3B* (29.6%; 209/707), equivalent to CNR1 as described by Niederer et al. [38]. Within CNR1, one or more copies were deleted in 61/209 individuals (29%) and duplicated in 148/209 individuals (71%). Thus, in the total group of 707 South African children, 8.6% carried a CNR1 deletion and 20.9% a CNR1 duplication. We observed low variation within CNR2, which encompasses the complete *FCGR3A* and exons 1 to 6 of *FCGR2C* (1.7%; 12/707; 4 deletions and 8 duplications). In seven (1%) individuals, CNV in *FCGR2C*, *FCGR3A* and *FCGR3B* was observed simultaneously, with one deletion and six duplications. Deletion or duplication of *FCGR3A* alone was noted in 16 individuals (2.3%), 11 with a gene deletion and 5 with a gene duplication (Fig 1C).

Copy number variation in *FCGR2C* and *FCGR3B* were separately observed in 233/707 (33%) and 219/707 (31%) children, respectively, and did not associate with HIV-1 acquisition (*P* > 0.05; Table 2). Complete absence of *FCGR2C* and *FCGR3B* was observed in one HIV-1 infected child. *FCGR3A* showed low frequency in copy number variation in 33/707 (4.7%) individuals, with 16 (2.3%) carrying a single gene copy and 17 (2.4%) having three gene copies. No individual with complete absence of *FCGR3A* was observed. A significant difference in *FCGR3A* copy number distribution was observed between the HIV-1 infected and exposed-uninfected children. Using one *FCGR3A* copy as reference, gene duplication was independently associated with increased odds of HIV-1 acquisition (OR = 10.27; 95% CI 2.00–52.65; *P* = 0.005, *P*_Bonf_ = 0.03; Table 2).

**Table 2:** Associations of *FCGR* variants with perinatal HIV-1 acquisition.

Variants	HIV-1-exposed uninfected	HIV-1 infected	OR (95% CI)	*P* value
	n = 312	n = 395		
*FCGR2C* copy number				
** **≤ 1 copy	31 (10)	37 (9)	Ref	
** **2 copies	209 (67)	265 (67)	1.06 (0.64–1.77)	0.816
** **≥ 3 copies	72 (23)	93 (24)	1.08 (0.61–1.91)	0.785
*FCGR3A* copy number				
** **1 copy	11 (3.5)	5 (1.3)	Ref	
** **2 copies	298 (95.5)	378 (95.2)	2.78 (0.95–8.08)	0.061
** **3 copies	3 (1.0)	14 (3.5)	10.27 (2.00–52.65)	**0.005 (*P***_**Bonf**_ = **0.03)**
*FCGR3B* copy number				
** **≤ 1 copy	28 (9)	35 (9)	Ref	
** **2 copies	217 (70)	271 (69)	0.99 (0.59–1.69)	0.997
** **≥ 3 copies	67 (21)	89 (22)	1.06 (0.59–1.92)	0.840
*FCGR2A* genotype				
** **166HH	65 (20.8)	86 (21.8)	Ref	
** **166HR	154 (49.4)	187 (47.3)	0.92 (0.62–1.35)	0.663
** **166RR	93 (29.8)	122 (30.9)	0.99 (0.65–1.51)	0.968
Allele carriage				
** **≥ 1 166H allele	219 (70)	273 (70)	0.95 (0.69–1.31)	0.757
** **≥ 1 166R allele	247 (79)	309 (78)	0.95 (0.66–1.36)	0.762
*FCGR2B* genotype				
** **232II	147 (47)	165 (41.8)	Ref	
** **232IT	126 (40)	160 (40.5)	1.13 (0.82–1.56)	0.453
** **232TT	39 (13)	70 (17.7)	1.60 (1.02–2.51)	**0.041** (*P*_Bonf_ = 0.246)
Allele carriage				
** **≥ 1 232I allele	273 (88)	325 (82)	0.66 (0.43–1.01)	0.057
** **≥ 1 232T allele	165 (53)	230 (58)	1.24 (0.92–1.67)	0.156
*FCGR3A* genotype				
** **176FF	134 (43)	155 (39)	Ref	
** **176FV	135 (43)	176 (45)	1.13 (0.82–1.56)	0.467
** **176VV	43 (14)	64 (16)	1.29 (0.82–2.02)	0.273
Allele carriage				
** **≥ 1 176F allele	269 (86)	331 (84)	0.83 (0.54–1.26)	0.373
** **≥ 1 176V allele	178 (57)	240 (61)	1.17 (0.86–1.58)	0.319
*FCGR3B* genotype				
** **HNA1a+/1b+/1c-	101 (32)	116 (29.37)	Ref	
** **HNA1a+/1b+/1c+	13 (4)	16 (4.05)	1.07 (0.49–2.34)	0.862
** **HNA1a+/1b−/1c+	44 (14)	61 (15.44)	1.21 (0.75–1.93)	0.433
** **HNA1a+/1b-/1c-	60 (19)	98 (24.81)	1.42 (0.94–2.16)	0.098
** **HNA1a-/1b+/1c+	43 (14)	50 (12.66)	1.01 (0.62–1.65)	0.960
** **HNA1a-/1b+/1c-	28 (9)	38 (9.62)	1.18 (0.68–2.06)	0.556
** **HNA1a-/1b-/1c+	23 (7)	15 (3.80)	0.57 (0.28–1.15)	0.115
** **HNA1a-/1b-/1c-	0 (0)	1 (0.25)	-	-
Allele carriage				
** **≥1 HNA1a allotype	218 (70)	290 (73)	1.19 (0.86–1.66)	0.298
** **≥1 HNA1b allotype	185 (59)	221 (56)	0.87 (0.65–1.18)	0.372
** **≥1 HNA1c allotype	123 (39)	141 (36)	0.85 (0.63–1.16)	0.309

Data are expressed as n (%).

OR, Odds Ratio; CI, Confidence Interval; *P*_Bonf_, Bonferroni corrected *P* value.

Bold indicates statistical significance of *P <* 0.05.

### *FCGR2A* and *FCGR3A* genotypes did not associate with perinatal HIV-1 acquisition

For the FcγRIIa-H166R genotype, 341 (48.2%) children were heterozygous (FcγRIIa-166HR), 151 (21.4%) were homozygous for the higher affinity IgG binding allele (FcγRIIa-166HH) and 215 (30.4%) homozygous for FcγRIIa-166RR. The genotype distributions of the *FCGR3A* were 311 (44%) for FcγRIIIa-176FV heterozygotes, 289 (41%) for FcγRIIIa-176FF, and 107 (15%) for FcγRIIIa-176VV. The FcγRIIa and FcγRIIIa genotype distributions observed in this study are similar to previous findings [12, 41]. FcγRIIa-H166R and FcγRIIIa-V176F genotype and allele carriage frequencies did not differ significantly between the HIV-1 infected and uninfected cohort (Table 2). Neither genotypes significantly associated with HIV-1 acquisition in the univariate or multivariate analyses (*P* > 0.05 for all comparisons).

### Associations between *FCGR2B* and *FCGR3B* genotypes and perinatal HIV-1 acquisition

The FcγRIIb-232II was the most prevalent *FCGR2B* genotype (44.1%; n = 312), followed by 232IT (40.5%; n = 286) and 232TT (15.4%; n = 109). Homozygosity for the FcγRIIb-232T allele was overrepresented in the HIV-1-infected children compared to the exposed-uninfected children (17.7% vs. 13%; Table 2). Compared to the FcγRIIb-232II genotype, the FcγRIIb-232TT genotype significantly associated with increased odds of HIV-1 acquisition in univariate analysis (OR = 1.60; 95% CI 1.02–2.51; *P* = 0.041, *P*_Bonf_ > 0.05). At the *FCGR3B* locus, HNA1a was the dominant allotype (72%; n = 508), followed by HNA1b (57%; n = 406) and HNA1c (37%; n = 264). We observed an overrepresentation of homozygous FcγRIIIb-HNA1a allotype in HIV-1-infected children compared to the exposed-uninfected (24.81% vs. 19%) but it did not independently associate with HIV-1 acquisition (OR = 1.42; 95% CI 0.94–2.16; *P* = 0.098, *P*_Bonf_ > 0.05; Table 2).

The association with homozygous FcγRIIIb-HNA1a attained significance after further assessment in a multivariate model that controlled for *FCGR3A* copy number and *FCGR2B* genotype, which were independently associated with HIV-1 acquisition (AOR = 1.55; 95% CI 1.01–2.38; *P* = 0.044, *P*_Bonf_ > 0.05). Both *FCGR3A* copy number (AOR = 10.68; 95% CI 2.04–55.86; *P* = 0.005, *P*_Bonf_ = 0.03) and *FCGR2B* genotype (AOR = 1.72; 95% CI 1.07–2.76; *P* = 0.024, *P*_Bonf_ > 0.05) remained significant (Table 3). The strength of association for *FCGR2B* genotype increased (AOR = 2.28; 95% CI 1.11–4.69; *P* = 0.024, *P*_Bonf_ > 0.05) when adjusted for *FCGR2C* c.134-96C>T that associated with HIV-1 acquisition in our previous study [42]. We further explored the associations in a subset of the study cohort that excluded breastfed infants (91 HIV-1 infected and 22 HIV-1 exposed-uninfected; nested total n = 586) and controlled for birthweight. The FcγRIIb-232TT genotype (AOR = 1.83; 95% CI 1.13–2.97; *P* = 0.014, *P*_Bonf_ > 0.05), homozygous FcγRIIIb-HNA1a allotype (AOR = 1.66; 95% CI 1.07–2.57; *P* = 0.025, *P*_Bonf_ > 0.05) and *FCGR3A* copy number (AOR = 8.58; 95% CI 1.60–45.92; *P* = 0.012, *P*_Bonf_ > 0.05) retained significance (Table 4).

**Table 3:** Multivariate analysis of the effect of *FCGR3A* copy number, *FCGR2B* and *FCGR3B* variants on perinatal HIV-1 acquisition.

Variants	Adjusted OR (95% CI)*	*P* value
*FCGR3A* copy number		
1 copy	Ref	
2 copies	2.81 (0.94–8.36)	0.064
3 copies	10.68 (2.04–55.86)	**0.005 (*P***_**Bonf**_ = **0.03)**
*FCGR2B* genotype		
232II	Ref	
232IT	1.20 (0.86–1.67)	0.295
232TT	1.72 (1.07–2.76)	**0.024** (*P*_Bonf_ = 0.144)
*FCGR3B* genotype		
HNA1a+/1b+/1c-	Ref	
HNA1a+/1b+/1c+	1.18 (0.54–2.60)	0.674
HNA1a+/1b−/1c+	1.27 (0.78–2.05)	0.333
HNA1a+/1b-/1c-	1.55 (1.01–2.38)	**0.044** (*P*_Bonf_ = 0.264)
HNA1a-/1b+/1c+	1.02 (0.63–1.68)	0.917
HNA1a-/1b+/1c-	1.10 (0.63–1.95)	0.734
HNA1a-/1b-/1c+	0.64 (0.31–1.32)	0.228

OR, Odds Ratio; CI, Confidence Interval; *P*_Bonf_, Bonferroni corrected *P* value.

Bold indicates statistical significance of *P <* 0.05.

* Multivariate model controlled for *FCGR3A* copy number, *FCGR2B* and *FCGR3B* variants.

**Table 4:** Associations of *FCGR* variants with perinatal HIV-1 acquisition in non-breastfed children after adjusting for birthweight.

Variants	HIV-1-exposed uninfected	HIV-1 infected	Univariate		Multivariate*	
			OR (95% CI)	*P* value	Adjusted OR	*P* value
					(95% CI)	
	n = 289	n = 297				
*FCGR3A* copy number						
1 copy	11 (3.5)	5 (1.3)	Ref		Ref	
2 copies	298 (95.5)	378 (95.2)	2.78 (0.95–8.08)	0.061	2.49 (0.82–7.54)	0.107
3 copies	3 (1.0)	14 (3.5)	10.27 (2.00–52.65)	**0.005 (*P***_**Bonf**_ = **0.03)**	8.58 (1.60–45.92)	**0.012** (*P*_Bonf_ = 0.072)
*FCGR2B* genotype						
232II	140 (48.4)	123 (41.4)	Ref		Ref	
232IT	113 (39.1)	122 (41.1)	1.13 (0.82–1.56)	0.453	1.27 (0.90–1.80)	0.171
232TT	36 (12.5)	52 (17.5)	1.60 (1.02–2.51)	**0.041** (*P*_Bonf_ = 0.246)	1.83 (1.13–2.97)	**0.014** (*P*_Bonf_ = 0.084)
*FCGR3B* genotype						
HNA1a+/1b+/1c-	94 (32.53)	90 (30.30)	Ref		Ref	
HNA1a+/1b+/1c+	11 (3.81)	12 (4.38)	1.07 (0.49–2.34)	0.862	1.27 (0.56–2.84)	0.568
HNA1a+/1b−/1c+	42 (14.53)	40 (13.47)	1.21 (0.75–1.93)	0.433	1.32 (0.80–2.15)	0.275
HNA1a+/1b-/1c-	57 (19.72)	76 (25.59)	1.42 (0.94–2.16)	0.098	1.66 (1.07–2.57)	**0.025** (*P*_Bonf_ = 0.15)
HNA1a-/1b+/1c+	37 (12.8)	41 (13.8)	1.01 (0.62–1.65)	0.960	1.12 (0.59–1.63)	0.937
HNA1a-/1b+/1c-	26 (9)	26 (8.75)	1.18 (0.68–2.06)	0.556	0.95 (0.63–2.02)	0.676
HNA1a-/1b-/1c+	22 (7)	10 (3.37)	0.57 (0.28–1.15)	0.115	0.55 (0.30–1.32)	0.217
HNA1a-/1b-/1c-	0 (0)	1 (0.34)	-	-		

OR, Odds Ratio; CI, Confidence Interval; *P*_Bonf_, Bonferroni corrected *P* value.

Bold indicates statistical significance of *P <* 0.05.

* The multivariate analysis adjusted for all 3 genetic parameters (*FCGR3A* copy number, *FCGR2B* and *FCGR3B* genotypes) simultaneously plus birthweight.

### Linkage disequilibrium of functionally relevant variants in the *FCGR2/3* locus

**Fig 2:**
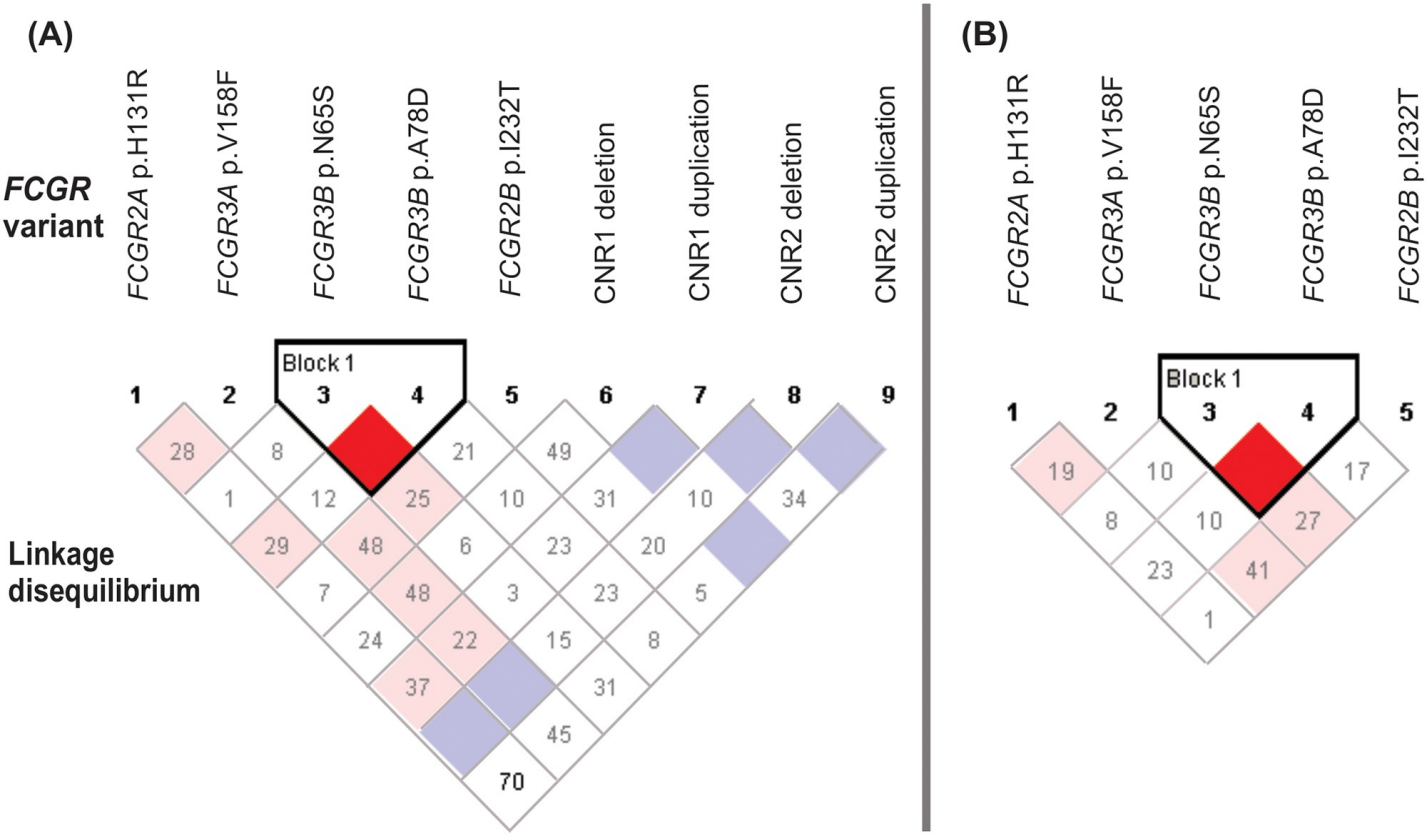
Linkage disequilibrium of functional FCGR variants in South African children born to women living with HIV-1. **(A)** All individuals, with or without CNV (n = 707); **(B)** only individuals with two gene copies (n = 474). The black triangle illustrates a haplotype block. Values reflect D′ measures of LD and colour in the squares given by standard D′ divided by log of the odds of LD between two loci (LOD). Bright red colour indicates very strong LD.

The functionally-relevant variants in the *FCGR2/3* locus have been reported to be in strong linkage disequilibrium due to physical proximity of the genes on chromosome 1q23 [43–45]. The observed genotype frequencies for FcγRIIa-H1166R, FcγRIIIa-V176F and FcγRIIIb-HNA1a/b/c were in Hardy-Weinberg equilibrium (*P* > 0.05) but those for FcγRIIb-I232T were not (*P* = 0.018). We assessed linkage disequilibrium between *FCGR2A*, *FCGR2B*, *FCGR3A and FCGR3B* variants, with and without considering the CNV, to determine whether the observed associations with independent *FCGR* variants are linked due to coinheritance of alleles at different loci. All participants were included irrespective of copy number; those with 3 or more copies were considered heterozygous if both alleles were present and homozygous if all copies carried the same allele. We found the FcγRIIIb-HNA1a/b/c haplotype in complete LD as expected (D′ = 1.0; r^2^ = 0.243). The FcγRIIb-I232T was in weak LD with FcγRIIIb-HNA1a/b (D′ = 0.254; r^2^ = 0.032) and FcγRIIIa-V176F (D′ = 0.486; r^2^ = 0.077). Similarly, weak LD was observed between FcγRIIa-H166R and FcγRIIIa-V176F (D′ = 0.280; r^2^ = 0.052), and the FcγRIIIb-HNA1c allotype (D′ = 0.297; r^2^ = 0.02). When only those with two gene copies were included, the observed LD pattern remained the same (Fig 2). Multivariate analysis was used to test allelic association for each variant that had some LD. The observed association remained significant for FcγRIIb-I232T (AOR = 1.69; 95% CI 1.06–2.70; *P* = 0.028, *P*_Bonf_ > 0.05) while FcγRIIIb-HNA1a did not (AOR = 1.50; 95% CI 0.98–2.30; *P* = 0.060) and remained not significant for FcγRIIa-H166R and FcγRIIIa-V176F.

## Discussion

The human *FCGR2/3* locus comprise activating and inhibitory receptors that are highly polymorphic (including SNPs and CNV), with functional implications. Whilst the role of some of the genetic variants are not well understood, the functional and clinical relevance of others in the pathogenesis of autoimmune and infectious diseases has been well documented [17, 18, 25, 45–47]. Furthermore, functional *FCGR* polymorphisms have been investigated in the context of HIV-1 acquisition [11, 12, 28, 42], disease progression [10, 20, 48, 49] and response to vaccination regimens [21, 22, 48] with inconsistent findings. In this study, we report further associations between the functional *FCGR* polymorphisms and HIV-1 acquisition in black South African children born to women living with HIV. The analysis excluded an association with *FCGR2C* variants, which was separately assessed by the authors in another study [42]. In that study, the *FCGR2C* c.134-96C>T tag variant produced a deleterious association in perinatal HIV-1 acquisition in contrast to the observed protective effect in the Thai RV144 vaccine trial [21].

The potential role of FcγR variants in modulating perinatal HIV-1 transmission and acquisition in South Africa was initially investigated by Lassaunière et al. [12], albeit in a small sample cohort. This present study used a larger cohort to validate the previously observed findings and determine if new FcγR variants associated with perinatal HIV-1 acquisition that may have been confounded by the earlier smaller sample size. The present study adds to a limited number of studies investigating the association between *FCGR* polymorphisms and HIV-1 acquisition in the maternal-infant HIV-1 transmission model. Whereas previous studies in Kenyan [11, 28] and South African [12] cohorts used data from mother-infant pairs, only infant data was available for our study cohort.

We did not observe an association between the common *FCGR2A* and *FCGR3A* polymorphisms and HIV-1 acquisition. This is in line with findings from independent Kenyan [28] and South African [12] cohorts, as well as a large genome-wide association study of adults [50], but contrasts the increased acquisition risk associated with FcγRIIa-166HH genotype reported in another Kenyan cohort [11]. Cohort differences, study design and statistical rigor employed have been suggested as possible reasons for the observed variable results [28, 50].

Other FcγR variants beyond FcγRIIa-H166R and FcγRIIIa-V176F, which include FcγRIIb-I232T, FcγRIIIb-HNA1a/b/c, and gene copy number are rarely studied in the context of HIV-1, in particular MTCT. Our earlier study [12] included FcγRIIb-I232T, and found that possession of at least one 232I allele was protective against in utero infection. Since this current study comprises a completely different cohort and are presumed to be predominantly in utero infected infants (since all received nevirapine at birth that reduces intrapartum transmission), our results on this larger cohort confirm those reported previously. Here we show that homozygosity for the FcγRIIb-232T minor allele associated with increased odds of perinatal acquisition of HIV-1. These findings suggest that the FcγRIIb-232TT genotype exerts a controlling influence on infant susceptibility to HIV-1 infection. FcγRIIb transmits signals via an immunoreceptor tyrosine-based inhibitory motif (ITIM) in its cytoplasmic tail. The FcγRIIb-232T polymorphism affects the receptor’s ability to translocate to lipid rafts, disrupting the inhibitory function of FcγRIIb, leading to a potentially higher activation state of cells [51, 52]. The *FCGR2B/C* promoter variants at position c.−386G>C and c.−120A>T also influence FcγRIIb expression but such an effect would not play a role in our cohort because African individuals do not possess the promoter variant in *FCGR2B* [43].

The FcγRIIb-232T and FcγRIIIb-HNA1a alleles are subject to ethnic variation, both being more prevalent in black compared to white South Africans [FcγRIIb-232T (30.9% vs. 10.9%; FcγRIIIb-HNA1a (50.6% vs. 36.2%)] [43]. The observed genotype frequencies for FcγRIIb-I232T were not in Hardy-Weinberg equilibrium possibly because of selection pressure from potent endemic infections in Africa, such as malaria [47]. Significant selection pressure is a likely driver of retention of the FcγRIIb-232T allele that produced a deleterious effect on susceptibility of HIV-1 infection in South African children.

Gene CNV not only contributes to differences in expression levels but also alters the cellular distribution of FcγRs in response to activation by IgG complexes [53]. Variation in copy number of *FCGR3A* has been shown to correlate with FcγRIIIa surface expression and function of NK cells [23]. In the present study, duplication or deletion of *FCGR3A* occurred either alone, in combination with *FCGR2C* or simultaneously with both *FCGR2C* and *FCGR3B* and significantly associated with HIV-1 acquisition. Specifically, we observed a trend towards an association of *FCGR3A* duplications but due to the low frequency of *FCGR3A* duplication, further studies of larger sample size are needed. We also observed 8.6% of the South African children carried a CNR1 deletion, which leads to the formation of *FCGR2C/2B* chimeric genes. This results in unusual expression of inhibitory FcγRIIb on NK cells and subsequently, reduced ADCC activity [24]. Although, this genotype did not associate with HIV-1 acquisition.

The FcγRIIIb is a glycosylphosphatidylinositol (GPI)-anchored protein, expressed largely on neutrophils [1]. Neutrophils from homozygous HNA1a individuals display higher affinity for IgG1 and IgG3 and greater phagocytic capacity than homozygous HNA1b individuals [27]. We observed an association between FcγRIIIb-HNA1a/b/c allotype and perinatally acquired HIV-1 infection. Specifically, homozygosity for the FcγRIIIb-HNA1a allotype produced a deleterious effect on perinatal HIV-1 acquisition. This is contrary to the protective effect observed in the earlier study with a smaller South African cohort, primarily in the intrapartum infected children [12]. The different observations between the two studies may be attributable to different cohort compositions. The present study cohort was exposed to nevirapine for prevention of MTCT and more likely infected in-utero, with few intrapartum infections. When the breast-fed children were excluded from the analysis, the observed significant association with FcγRIIb-232T, FcγRIIIb-HNA1a and *FCGR3A* copy number variants remained. These variants likely play a role in HIV-1 acquisition during the course of pregnancy and at the maternal-foetal interface [12].

The study has several strengths. The genotyping method utilized is robust, as the MLPA assay is able to assess functional SNPs and CNV within the *FCGR2/3* locus simultaneously, rather than investigating associations with perinatal HIV-1 infection using methodologies that use candidate gene designs. Due to high homology of *FCGR2/3* we checked for linkage disequilibrium to identify functional interaction between the independently associated polymorphisms. A limitation of the study is that maternal data were not available. In particular, we could not adjust for maternal viral load, a key determinant of MTCT of HIV-1 infection. Furthermore, we could not assess the the effect of maternal *FCGR* genotypes on transmission.

The contribution of FcγRIIb to disease susceptibility has largely been studied in systemic lupus erythematosus patients [47, 51] but there is paucity of data on association with HIV-1 acquisition. The findings of this study contribute to better understanding of the role of FcγRs in HIV-1 infection in children and add to the growing evidence of a potential role for Fc-mediated effector functions in modulating perinatal HIV-1 acquisition. As more FcγR variants associated with HIV-1 acquisition are reported, more studies are needed to critically evaluate their clinical relevance in the development of preventive or therapeutic interventions.

## Supporting information

S1 File Dataset for FCGR Variants and MTCT of HIV-1. (XLSX)

S2 File (DTA)
